# Comparative Effectiveness of Cognitive Therapies Delivered Face-to-Face or over the Telephone: An Observational Study Using Propensity Methods

**DOI:** 10.1371/journal.pone.0042916

**Published:** 2012-09-28

**Authors:** Geoffrey C. Hammond, Tim J. Croudace, Muralikrishnan Radhakrishnan, Louise Lafortune, Alison Watson, Fiona McMillan-Shields, Peter B. Jones

**Affiliations:** 1 National Institute for Health Research Collaboration for Leadership in Applied Health Research & Care for Cambridgeshire and Peterborough, Cambridge, United Kingdom; 2 Cambridgeshire & Peterborough National Health Service Foundation Trust, Cambridge, United Kingdom; 3 Department of Health Sciences and Hull York Medical School, University of York, York, United Kingdom; 4 Cambridge Institute of Public Health, Cambridge, United Kingdom; 5 National Health Service Midlands & East, Cambridge, United Kingdom; 6 Department of Psychiatry, University of Cambridge and Cambridgeshire & Peterborough National Health Service Foundation Trust, Cambridge, United Kingdom; Federal University of Rio de Janeiro, Brazil

## Abstract

**Objectives:**

To compare the clinical and cost-effectiveness of face-to-face (FTF) with over-the-telephone (OTT) delivery of low intensity cognitive behavioural therapy.

**Design:**

Observational study following SROBE guidelines. Selection effects were controlled using propensity scores. Non-inferiority comparisons assessed effectiveness.

**Setting:**

IAPT (improving access to psychological therapies) services in the East of England.

**Participants:**

39,227 adults referred to IAPT services. Propensity score strata included 4,106 individuals; 147 pairs participated in 1∶1 matching.

**Intervention:**

Two or more sessions of computerised cognitive behavioural therapy (CBT).

**Main outcome measures:**

Patient-reported outcomes: Patient Health Questionnaire (PHQ-9) for depression; Generalised Anxiety Disorder questionnaire (GAD-7); Work and Social Adjustment Scale (WSAS). Differences between groups were summarised as standardised effect sizes (ES), adjusted mean differences and minimally important difference for PHQ-9. Cost per session for OTT was compared with FTF.

**Results:**

Analysis of covariance controlling for number of assessments, provider site, and baseline PHQ-9, GAD-7 and WSAS indicated statistically significantly greater reductions in scores for OTT treatment with moderate (PHQ-9: ES: 0.14; GAD-7: ES: 0.10) or small (WSAS: ES: 0.03) effect sizes. Non-inferiority in favour of OTT treatment for symptom severity persisted as small to moderate effects for all but individuals with the highest symptom severity. In the most stringent comparison, the one-to-one propensity matching, adjusted mean differences in treatment outcomes indicated non-inferiority between OTT versus FTF treatments for PHQ-9 and GAD-7, whereas the evidence was moderate for WSAS. The per-session cost for OTT was 36.2% lower than FTF.

**Conclusions:**

The clinical effectiveness of low intensity CBT-based interventions delivered OTT was not inferior to those delivered FTF except for people with more severe illness where FTF was superior. This provides evidence for better targeting of therapy, efficiencies for patients, cost savings for services and greater access to psychological therapies for people with common mental disorders.

## Introduction

The programme to improve access to psychological therapies (IAPT) is the most significant development in English mental health services since the closure of the asylums and the advent of community care. Whilst those developments concerned severe mental illness and secondary care, IAPT targets mild to moderate depression and anxiety. These are common conditions seen frequently in general practice [1], [2]; they cause enormous disability at the population level [3], [4]. The financial cost of depression in the UK was estimated at approximately 150 billion pounds in 2009/2010, of which 30 billion is thought to be related to inability to work [1].

Predicated on economic arguments and clinical evidence, IAPT promotes access to talking treatments based on cognitive behavioural therapy (CBT) approved by the National Institute of Clinical Excellence (NICE). IAPT services solicit referrals (including self-referral) and reduce waiting times [1], [2] for CBT by substantially increasing the numbers of therapists. More than 300 new therapists were recruited between 2008 and 2011 in the East of England (EoE) alone. Maintaining or increasing the working capacity of patients are important secondary goals that underpin economic arguments for IAPT [1]. There are two tiers of IAPT therapy, depending on clinical severity, and corresponding to NICE steps 2 and 3 for the treatment of depression and anxiety. More intense therapy is delivered by more experienced clinicians in the higher tier. The present study concerns the lower tier that provides treatment for the majority of referrals from primary care and other sources.

Two IAPT demonstration sites in England (Newham and Doncaster) provided observational evidence that face-to-face (FTF) and over-the-telephone (OTT) delivery of psychological therapy were both effective in depression and anxiety [3]. Brief, CBT-based interventions led to significant reductions in symptoms of a magnitude similar to that reported in specialist out-patient psychotherapy services [4], [5]. These findings are in line with a randomised controlled trial of telephone CBT [6] and a recent non-inferiority trial comparing OTT with FTF interventions for obsessive compulsive disorder [7], [8].

Telephone mediated psychological interventions are convenient for patients and therapists, with a 40% reduction in treatment time [7], [8] and removal of barriers to treatment [9]. Services are no longer constrained by working hours or treatment space. However, the evidence for these benefits relies on small samples in specialised settings, and may not be relevant to the relatively brief interventions (fewer than six sessions) delivered in the lower tier of IAPT [10].

OTT may represent a cost-effective option for IAPT. Both OTT and FTF services have already been implemented by the National Health Service across England, preventing the assessment of comparative effectiveness of mode of delivery of the therapy through randomised designs at the individual or even cluster (therapist or service) level. Therefore, we employed a stepped approach to the analysis of observational data, attempting to minimise the disadvantages of a non-randomised design and maximise the information relevant to the assessment of comparative effectiveness of OTT and FTF therapy. We used patient-rated outcomes from 190,128 treatment sessions within IAPT sites in the EoE to compare the clinical and cost effectiveness of low intensity, CBT-based psychological interventions delivered OTT versus FTF.

## Methods

### Ethics Statement

The study design and database were reviewed by the National Research and Ethics Service (NRES) for England. NRES considered the work to be an evaluation of existing services using anonymised clinical record data and did not require further ethical review.

### Setting and participants

All subjects entered treatment after referral to IAPT services commissioned by seven primary care trusts (PCTs; organisations charged with commissioning care from health providers) in the EoE region from September 2008 until September 2010. These were: NE Herts, NE Essex, Suffolk, W Herts and Mid Essex, Bedfordshire, and Cambridgeshire. These are referred to in a different order as services A–G. All had been providing treatment for longer than 12 months at the time of data extraction; five remaining PCTs delivering IAPT services in the EoE had yet to achieve this stability and were excluded.

All services implemented a stepped-care model: patients could receive either high or low intensity interventions, as deemed appropriate by a standard initial assessment. We focused our analysis on patients treated exclusively with low intensity interventions where OTT is a treatment option for trained therapists working in accordance with service implementation guidelines [11]. Six low intensity interventions, alone or in combination, were approved for delivery either FTF or OTT, a decision taken by the therapist at assessment in collaboration with the patient; there were no operational guidelines. The low intensity interventions were computerised cognitive behavioural therapy (C-CBT), books on prescription or guided self-help, behavioural activation, structured physical exercise, or attendance at psycho-educational groups.

Individuals were excluded from the analysis if they:

- received or were scheduled to receive one or more high intensity treatments (defined as either receiving visits marked as high intensity and/or receiving treatments associated with a high intensity interventions);
- attended fewer than two sessions;
- attended fewer than two sessions in which the outcome measures (psychometric rating scales assessing depression, anxiety) were completed;
- had no recorded treatment outcome;
- had more than a single recorded session of behavioural activation or structured exercise.

For the cost-minimisation analysis, information about number and type of treatment sessions were extracted from the clinical data while IAPT cost information, also anonymised, was extracted from reports prepared by Mental Health Strategies for the Department of Health [12]. The evaluation was funded and supported by NHS East of England and the NIHR CLAHRC for Cambridgeshire & Peterborough. The design, analyses and drafting of this manuscript were undertaken in accordance with STROBE guidelines [13].

### Defining intervention groups: CBT delivered over-the-telephone (OTT) or face-to-face (FTF)

People referred to IAPT underwent a routine, baseline face-to-face assessment after which subjects were allocated to high or low intensity treatment, thereafter. The treatment comparison groups for this study were derived from those allocated to low intensity treatments who were themselves, divided between FTF therapy, where all subsequent sessions were face-to-face, and therapy given Over-The-Telephone OTT. This latter group includes a small number of people who did not receive the routine FTF baselines assessment because a single IAPT service provider arranged for this to be undertaken by the referrer (see Results); subjects in the OTT group received all subsequent therapy OTT regardless of where the initial assessment took place. Subjects who, following assessment, received a mixture of FTF and OTT therapies were excluded from the analysis.

### Patient-reported Outcomes (PROs)

Three PROs are mandated for use in patient evaluation as per IAPT implementation guidelines [11]. They are measured at baseline assessment and before each subsequent treatment session. Patients complete the three outcome measures themselves, either on paper or on screen, as they prefer. The scores are available for discussion between the therapist and patient during the subsequent treatment session, whether OTT or FTF. Neither therapists nor patients were aware that the current comparative effectiveness study would take place.

#### The Patient Health Questionnaire Depression scale (PHQ-9)

Nine questions assess symptoms of depression and are scored from 0 (“Not at all bothered by the problem”) to 3 (“Bothered nearly every day”). Sum scores range from 0 to 27. A score of 10 has been suggested as a cut point for a clinical diagnosis of depression [14]; severity bands for symptoms levels in terms of PHQ-9 scores are shown later in Results, Table 1.

**Table 1:** Baseline demographic, clinical, and functional characteristics of patients receiving either face-to-face or predominantly telephone based treatments.

Variable		Face-to-Face therapy (FTF) (n = 1791)		Over-the Telephone therapy (OTT) (n = 2315)	
		N	%	N	%
Age in years*	<18–25	274	15.3	384	16.6
	26–35	437	24.4	532	23.0
	36–45	444	24.8	591	25.5
	46–55	373	20.8	417	18.0
	56–65	209	11.7	265	11.4
	66–85	56	3.1	127	5.5
Gender	Female	1144	63.8	1526	65.9
Employment*	Full-Time	838	46.7	1037	44.8
	Part-Time	327	18.2	444	19.2
	Unemployed – seeking or occupied	310	17.3	321	13.9
	Inactive	318	17.7	514	22.2
Benefits/Sick pay	Receiving at baseline	416	23.2	497	21.5
Period of time in	<3 Months	93	5.2	31	1.3
mental health	3–6 Months	87	4.9	96	4.1
services at first	7–9 Months	84	4.7	170	7.3
IAPT session	10–12 Months	55	3.1	182	7.9
	12+ Months	1474	82.2	1837	79.3
Psychotropic medication*	Use at baseline	966	53.9	1167	50.4
Referral source*	GP referral	1675	93.4	1874	80.9
	Self-referral	41	2.3	238	10.3
	Any specialty	31	1.7	127	5.5
	Other	46	2.6	77	3.3
PCT*	A	53	3.0	755	32.6
	B	672	37.5	21	0.9
	C	247	13.8	33	1.4
	D	191	10.7	886	38.3
	E	534	29.8	90	3.9
	F	53	3.0	312	13.5
	G	43	2.4	219	9.5
PHQ-9 score*	No Impairment (0–5)	212	11.8	247	10.7
	Mild Impairment (5–9)	408	22.8	499	21.5
	Moderate Impairment (10–14)	455	25.4	681	29.4
	Moderate-Severe Impairment (15–19)	422	23.5	567	24.5
	Severe Impairment (20–27)	296	16.5	322	13.9
GAD-7 score	No Impairment (0–5)	193	10.8	223	9.6
	Mild Impairment (5–9)	451	25.2	594	25.6
	Moderate Impairment (10–14)	530	29.6	708	30.6
	Severe Impairment (15–21)	619	34.5	791	34.2
		**Mean**	**SD**	**Mean**	**SD**
WSAS score		16.4	9.1	15.2	8.9
Phobia Measures	Phobia Q1*	2.9	2.5	2.6	2.4
	Phobia Q2*	2.5	2.7	2.1	2.5
	Phobia Q3	2.2	2.7	2.1	2.6

* : Significant differences across treatment groups.

#### The Generalised Anxiety Disorder scale (GAD-7)

Seven items measure the severity of anxiety symptoms [15] using the same response options and item scores as the PHQ-9. Sum scores range from 0 to 21. A score of 8 or higher on the GAD-7 has been suggested as a threshold for a likely diagnosis of clinical anxiety [15]. Severity bands applicable to GAD-7 scores are shown in Table 1.

#### The Work and Social Adjustment Scale (WSAS)

Five self-report items regarding ability to work, home management, social leisure, private leisure and close relationships are each scored 0–8; zero indicates no impairment and eight is very severe impairment. The total score assesses overall functional impairment [16]. Severity bands are: 0–10 subclinical impairment; 10–20 functional impairment; 20–30 moderately severe impairment; 30+ severe impairment.

### Analytical Approaches

We used three approaches to analyse these observational data.

The first approach involved naïve comparisons between PROs for FTF and OTT treatments (unadjusted and adjusted, as described below). The second and third approaches used sampling methods based on propensity scores. This allowed us to adjust for potential confounding in the non-randomised design, particularly concerning selection by assessing clinicians of patients with certain characteristics to either FTF or OTT. These propensity methods allowed “matched” non-inferiority comparisons across the two groups using the patient-reported outcomes: symptom reduction (depression and anxiety), work and social adjustment. A cost-minimisation analysis based on treatment duration and previous estimates of per session costs was used to compare the cost implications of OTT and FTF sessions.

#### Approach 1: Naïve treatment comparisons

As a first step, differences in FTF versus OTT treatment effectiveness were assessed using an unadjusted ANCOVA model with only treatment as a factor. A second adjusted model controlling for baseline symptom severity (as measured using PHQ9, GAD7, and WSAS baseline scores), PCT, and treatment duration (represented as the number of attended sessions) was then developed.

#### Approaches 2 & 3: Propensity Score Development

In observational, non-randomised evaluations, naïve comparisons between individuals who receive different treatments (FTF vs. OTT) are confounded; treatment choice is influenced by an individual's baseline clinical, social, or behavioural factors that may themselves affect the outcome, independent of treatment allocation. A propensity score approach attempts to mitigate this selection bias by balancing as many observed covariates (which potentially influence selection to treatment) as possible across treatments, increasing the validity of comparisons between non-randomised treatment groups.

In this analysis, the propensity score represents the probability of receiving OTT rather than FTF treatment conditional on covariates entered into a logistic regression model. Covariates included demographic indicators (age and gender), baseline measures of symptom severity, work, social adjustment, and service level predictors. Three binary coded items assessing the presence of general or specific phobias were also included. Employment, benefit status, receipt of psychotropic medication at baseline, and referral source was also included. PCT (indicating a particular IAPT service or set of commissioned services) was included in the model to ensure that treatment selection was not biased by differences in treatment effectiveness or policy regarding OTT use. Other service characteristics entered included referral source and also how long the service had been operating when the patient was seen.

Individuals receiving different treatments with similar propensity scores can be considered matched and their outcomes can be directly compared. Unlike randomised designs, the assumption that covariates included in the propensity model largely account for treatment selection requires careful examination of regression results.

Given that baseline characteristics were routinely assessed but not always systematically recorded (lack of recording occurred in approximately 15% of cases), missing baseline data were imputed using corresponding variables collected at the second visit so as to increase the available analysis sample.

### Deriving the matched on propensity score samples for comparison

The two separate propensity score matching approaches used probability estimates resulting from the model. In Approach 2, patients were assigned to one of five strata based on probability estimates such that propensity for OTT vs. FTF was minimal within strata and maximal between. Those in the first stratum were similar to individuals receiving OTT interventions; those in the fifth stratum were similar to individuals receiving FTF treatment (see Results). Effectiveness of the OTT versus FTF intervention was compared between treatments within each stratum.

Approach 3 used nearest-neighbour, 1∶1, non-replacement matching to assess the sensitivity of the stratification approach. Each individual receiving OTT treatment was matched to an individual receiving FTF treatment with a near equivalent propensity score. Once matched, both individuals were removed from the sample and the process repeated until no further pairs could be matched. This process produced two samples with identical sample sizes matched on propensity score values, but excluding a large number of unmatched individuals.

The 1∶1 matching was conducted within each of the seven PCTs to control for differences in the magnitude of the symptom change and differences in the balance of OTT and FTF implementation across providers. To ensure that services with a larger treatment volume or more matches did not bias the results, each service contributed the same number of matched pairs to the sample (n = 21 pairs; see Results), including all the matched pairs from the PCT with the smallest number. For the remainder, we drew random sub-samples with the same number of pairs (n = 21 pairs). Results were verified by repeatedly re-sampling the matched pairs with replacement.

The level of tolerable difference in 1∶1 matching was specified *a priori* using a value for the calliper estimate which determines the width of the propensity score interval. We adopted a conservative value of the logit (0.2 standard deviations).

### Statistical comparisons

To compare baseline and treatment characteristics between OTT and FTF, independent sample t-tests were used for differences in continuous outcomes. Chi-square or Fisher's exact tests were used for categorical/dichotomous variables. Within each stratum and 1∶1 matching sample, a random-effects ANCOVA model using the same covariates in the adjusted comparisons was used to compare the two treatments in terms of the three PROs: PHQ-9, GAD-7 and WSAS. Effect size (ES) estimates (Cohen's *d*) were provided with all OTT and FTF comparisons. Using established guidelines, ES values less than 0.10 were considered small, 0.11 to 0.25 were considered moderate, and those above 0.25 were considered large.

To assess non-inferiority between the treatments we used two-sided significance tests and 95% confidence intervals for score differences between treatment groups on all three outcome measures. The lower limit of the confidence interval (LCL) represents a boundary of non-inferiority. For all three measures, the LCLs were compared with small (0.2 x pooled S.D.) and medium (0.5 x pooled S.D.) estimates of statistical uncertainty. For the PHQ-9 depression scale, the minimally important difference estimate of 5 units was an additional measure with which to assess non-inferiority and the importance of any differences between treatments [17].

### Assessing cost-effectiveness

Data on session volume and corresponding total spend was available for five of the seven PCTs for financial year 2009/2010, allowing us to estimate OTT and FTF session costs. A cost-minimisation approach was selected on the basis of treatment equivalence [18]. The reported total spend on IAPT in each PCT was divided into a cost for low and high intensity activity. Initial micro-costing data indicated low intensity activity was 1.8 times less expensive than high intensity; representative but anonymous data from a PCT are available from the authors. A local tariff developed for IAPT services in the EoE region also confirmed that the cost ratio was closer to this estimate [19]. The proportion of low intensity sessions were adjusted for this base-case cost ratio of 1.8, providing a cost estimate of all low intensity activity. The 1.8 ratio was also varied from 1.2 to 2.0 to test the assumption's sensitivity.

Literature reports indicated that OTT requires shorter treatment durations than FTF [8], [9]. To derive the cost per OTT and FTF session the difference in session duration was assumed to directly translate into an equivalent difference in cost. We used an observed ratio of 1.5 for the difference in treatment duration observed between FTF and OTT to calculate cost per session. The estimated total cost for all low intensity sessions was apportioned based on the proportion of OTT and FTF sessions for each PCT, adjusting for the 1.5 ratio to arrive at the total cost of OTT and FTF. Dividing the total costs by the number of sessions provided session costs for each treatment.

## Results

### Patient flow, follow-up, and sample characteristics

**Figure 1:**
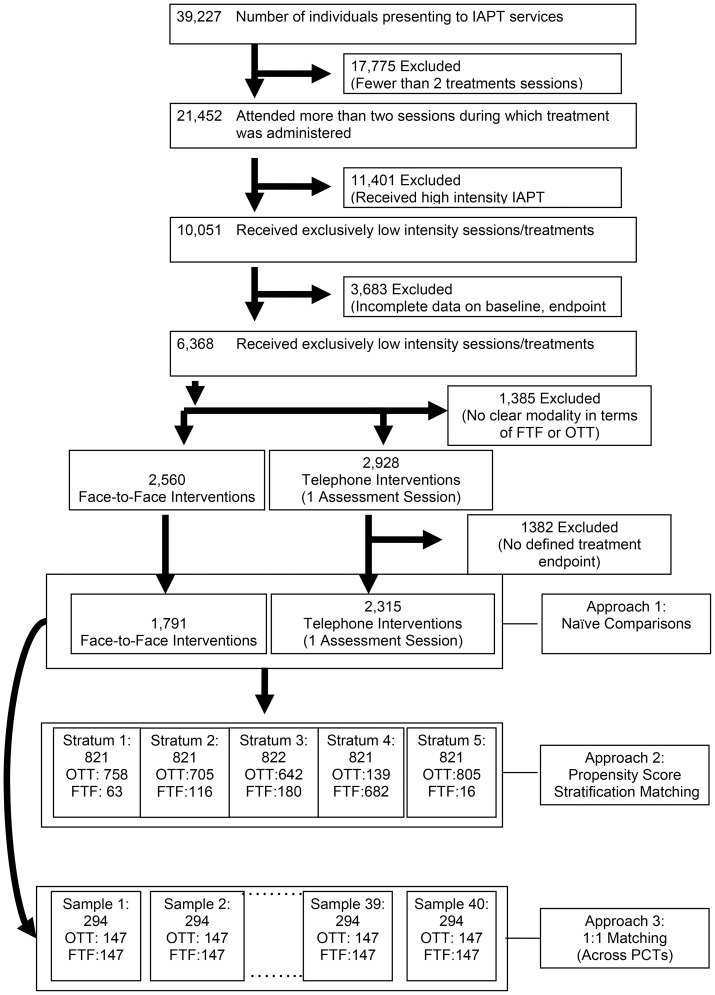
Flow and selection of subject records for inclusion into naïve comparisons and propensity score analyses.

Patient flow is described in Figure 1. During the survey period, 39,227 individuals were referred to IAPT services in seven PCTs. Of those, 21,452 (55%) attended at least two sessions during which treatment was administered: 11,401 (53%) of those received or were scheduled to receive a high intensity intervention, leaving 10,051 (47%) individuals receiving solely low intensity interventions. Of these people allocated to low intensity interventions, 6,873 (68%) had information on baseline and endpoint measures and all baseline covariates required for propensity score matching and calculation of change scores. These were the potential participants in this study.

The 6,873 potential participants were then divided into three groups based on receipt of OTT and/or FTF therapy:

1. *The FTF group*: 2560 people (32.3%) received exclusively FTF therapy; of these, 1791 (70%) had completed treatment at the time of data extraction and comprise the FTF group.
2. *The OTT group*: 2928 (46.0%) received only OTT interventions after a baseline FTF assessment (this included 311 people from a single IAPT service who received their baseline FTF assessment by the referrer, not that IAPT service); of these, 2315 (79%) had completed treatment at the time of data extraction and comprise the OTT group.
3. The remaining 1385 (21.7%) participants received a blend of OTT and FTF interventions and could not be reliably allocated to either treatment group; this third group of people receiving low-intensity interventions were excluded from our analyses.

#### Differences in baseline attributes across treatment groups

Table 1 displays baseline characteristics for both treatment groups before propensity score matching. The FTF group was more likely to be unemployed, referred by a general practitioner (family medical practitioner; GP) and to be receiving psychotropic medication at baseline. More people received FTF in PCTs B, C, and E. Individuals receiving FTF were also more likely to report moderately-severe or severe scores on the PHQ-9 and to have slightly higher average WSAS scores. Those who received OTT interventions were more likely to be economically inactive (e.g. student or homemaker). OTT interventions were more likely to have been received in PCTs A, D, and F. Small but significant differences were observed across groups in terms of age distribution, active service duration at first visit, and phobia questions 1 and 2.

### Comparison of outcomes for CBT delivered OTT versus FTF

Propensity score estimates (odds of OTT vs. FTF) are shown in Table 2. Older age was associated with FTF treatment. Individuals were less likely to receive OTT interventions if they were unemployed. The longer an IAPT service had been in operation, the more likely OTT interventions were to be used. Receipt of OTT treatment was less likely if the individual was seen in PCTs B, C, D and E relative to the reference provider (A). The model R-square was 0.50, indicating excellent predictive accuracy.

**Table 2:** Logistic regression of OTT versus FTF generating estimates and standard errors for variables included in the propensity score.

Variable		Estimate	S.E.	95% confidence		T-test	p-value
				Lower	Upper		
Age in years	<18–25	****					
	26–35	−0.18	0.17	−0.50	0.15	−1.06	0.29
	36–45	−0.19	0.17	−0.52	0.14	−1.14	0.25
	46–55	−0.47	0.17	−0.81	−0.12	−2.68	0.01
	56–65	−0.34	0.20	−0.73	0.05	−1.72	0.09
	66–85	−0.53	0.28	−1.08	0.01	−1.92	0.06
Gender	Female	0.02	0.11	−0.20	0.24	0.21	0.84
Employment	Full-Time	****					
	Part-Time	0.14	0.15	−0.15	0.42	0.94	0.35
	Unemployed – seeking or occupied	−0.42	0.16	−0.74	−0.11	−2.61	0.01
	Inactive	0.07	0.15	−0.23	0.37	0.44	0.66
Benefits/Sick pay	Receiving at baseline	−0.20	0.14	−0.48	0.07	−1.46	0.14
Period of time in	<3 Months	****					
mental health	3–6 Months	0.01	0.37	−0.72	0.74	0.03	0.98
services at first	7–9 Months	0.65	0.36	−0.06	1.35	1.79	0.07
IAPT session	10–12 Months	1.02	0.38	0.29	1.76	2.72	0.01
	12+ Months	2.04	0.44	1.17	2.90	4.62	<0.01
Psychotropic medication	Use at baseline	−0.07	0.10	−0.27	0.13	−0.67	0.50
Referral source	GP referral	****					
	Self-referral	0.22	0.21	−0.20	0.63	1.02	0.31
	Any specialty	0.25	0.34	−0.41	0.91	0.74	0.46
	Other	−0.51	0.29	−1.08	0.06	−1.75	0.08
PCT	A	****					
	B	−6.19	0.27	−6.71	−5.66	−23.0	<0.01
	C	−3.36	0.36	−4.06	−2.66	−9.39	<0.01
	D	−1.09	0.17	−1.43	−0.76	−6.44	<0.01
	E	−4.51	0.19	−4.89	−4.13	−23.5	<0.01
	F	0.56	0.41	−0.24	1.36	1.38	0.17
	G	0.15	0.39	−0.62	0.91	0.38	0.70
PHQ-9 score	No Impairment (0–5)	****					
	Mild Impairment (5–9)	−0.02	0.20	−0.42	0.37	−0.12	0.91
	Moderate Impairment (10–14)	−0.01	0.22	−0.43	0.41	−0.05	0.96
	Moderate-Severe Impairment (15–19)	−0.13	0.24	−0.60	0.33	−0.57	0.57
	Severe Impairment (20–27)	−0.37	0.26	−0.89	0.15	−1.40	0.16
GAD-7 score	No Impairment (0–5)	****					
	Mild Impairment (5–9)	−0.05	0.20	−0.45	0.35	−0.24	0.81
	Moderate Impairment (10–14)	−0.19	0.21	−0.61	0.22	−0.91	0.36
	Severe Impairment (15–21)	−0.10	0.23	−0.55	0.34	−0.45	0.66
WSAS score	No Impairment (0–10)	****					
	Mild Impairment (11–20)	−0.18	0.13	−0.44	0.08	−1.32	0.19
	Moderate Impairment (21–30)	−0.21	0.16	−0.53	0.10	−1.32	0.19
	Severe Impairment (31–40)	−0.12	0.26	−0.63	0.38	−0.47	0.64
Phobia measures	Phobia Q1	−0.03	0.02	−0.07	0.02	−1.16	0.25
	Phobia Q2	−0.01	0.02	−0.05	0.04	−0.32	0.75
	Phobia Q3	0.00	0.02	−0.04	0.04	0.11	0.92

Note: Values in bold indicate significant associations (p<.01).

#### Approach 1: Naïve Comparisons

**Figure 2:**
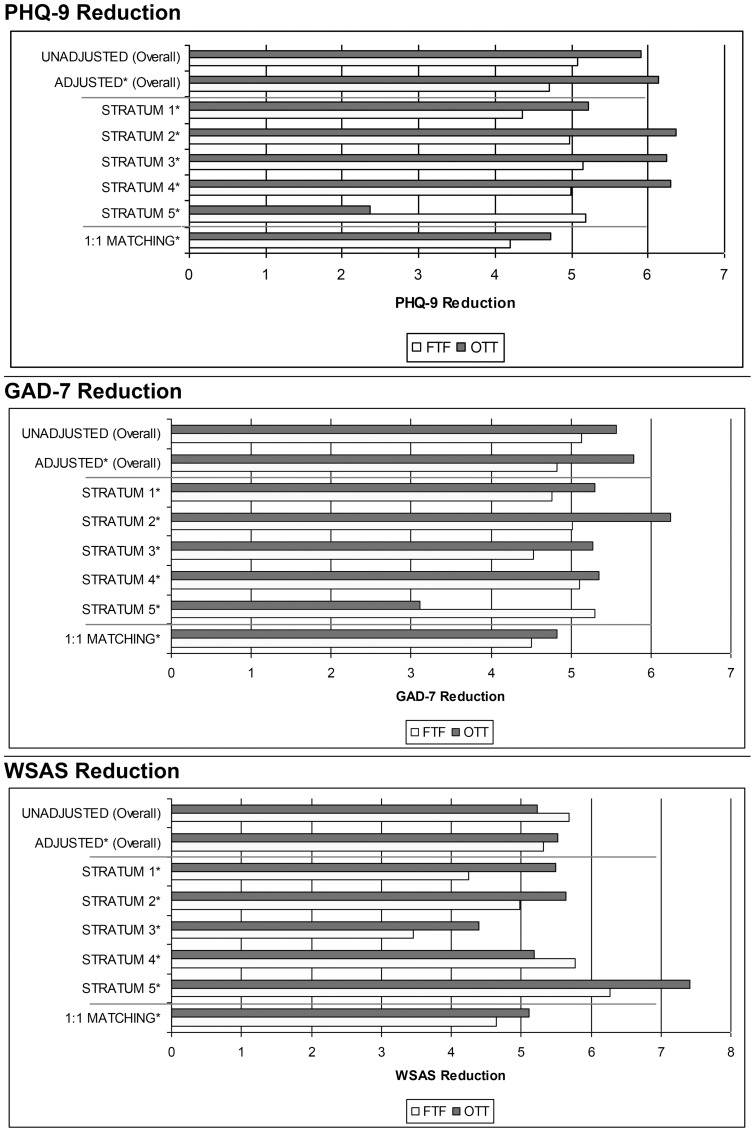
Average reductions in PHQ-9, GAD-7 and WSAS scores for FTF versus OTT therapy groups. Reductions in PHQ-9, GAD-7 and WASAS are each shown in a different pane. Comparisons between the reductions in the FTF and OTT groups shown by paired bars; OTT is always the upper and FTF the lower. These are presented first for the overall sample (adjusted and unadjusted; the first, naïve approach to analysis), then for the five propensity strata, and last for the 1∶1 matching procedure. Other than the first, unadjusted reductions, all average reductions (in the strata comparisons and in the 1∶1 matching) are adjusted for service provider, number sessions administered, and baseline symptom severity for the particular measure (PHQ9, GAD7 or WSAS). See: http://www.psychiatry.cam.ac.uk/wp-content/uploads/2012/08/PONE-D-11-20688-Figures.doc for colour version.

Figure 2 displays the mean reduction in PHQ-9, GAD-7 and WSAS for both OTT and FTF treatments in the overall sample (unadjusted and adjusted for provider, number of treatment sessions), each propensity stratum, and the 1∶1 matching sample. An unadjusted comparison shown in the top section indicated significant differences between treatments in the reductions of PHQ-9 and GAD-7 symptom scores; OTT interventions appeared to be more effective (PHQ-9: F = 17.5, p<.001, effect size (ES) = 0.13; GAD-7: F = 5.93, p = 0.015, ES = 0.07; WSAS: F = 2.82, p = 0.087, ES = 0.04). Significant differences between treatment groups on both symptom measures were still observed after controlling for number of assessments, provider sites, and baseline symptom severity measures (PHQ-9: F = 10.9, p<.001, ES = 0.14, GAD-7: F = 8.13, p = 0.042, ES = 0.10, WSAS: F = 3.12, p = 0.078, ES = 0.03).

#### Approach 2: Propensity Scores & Stratification Approach

Five propensity strata were developed using estimated propensity scores. This approach minimizes differences in propensity to being prescribed OTT vs. FTF by restricting the treatment comparison to subjects within each stratum where these propensity characteristics are similar (Table 3). There were 821 individuals in all strata except the third, in which there were n = 822. The number of individuals receiving OTT interventions in each stratum were 758 (93.6%) in the first stratum, 705 (88.5%) in the second, 642 (80.1%) in the third, 139 (17.3%) in the fourth, and 16 (2.0%) in the fifth. Strata differed markedly in their demographic, baseline symptom, and service characteristics, these factors being related to the odds of receiving OTT vs. FTF treatment.

**Table 3:** Patient characteristics in the five propensity score strata.

		Propensity Stratum					
		One n = 821	Two n = 821	Three n = 822		Four n = 821	Five n = 821
		n (%)					
Age in years	<18–25	175 (21.3)	111 (13.5)		117 (14.2)	146 (17.8)	109 (13.3)
	26–35	180 (21.9)	206 (25.1)		176 (21.4)	191 (23.3)	216 (26.3)
	36–45	230 (28.0)	212 (25.8)		183 (22.3)	211 (25.7)	198 (24.1)
	46–55	110 (13.4)	144 (17.5)		195 (23.7)	150 (18.3)	191 (23.3)
	56–65	87 (10.6)	94 (11.4)		105 (12.8)	89 (10.8)	98 (11.9)
	66–85	39 (4.8)	54 (6.6)		46 (5.6)	34 (4.1)	9 (1.1)
Gender	Female	583 (71.0)	517 (63.0)		523 (63.6)	529 (64.4)	517 (63.3)
Employment	Employment- Full-Time	377 (45.9)	393 (47.9)		332 (40.4)	375 (45.7)	397 (48.4)
	Employment- Part-Time	183 (22.3)	158 (19.2)		126 (15.3)	165 (20.1)	139 (16.9)
	Unemployed – seeking or occupied	63 (7.7)	91 (11.1)		193 (23.5)	118 (14.4)	165 (20.1)
	Inactive	198 (24.1)	179 (21.8)		171 (20.8)	163 (19.9)	120 (14.6)
Benefits/Sick pay	Receiving at baseline	43 (9.4)	69 (16.6)		69 (22.9)	85 (14.8)	143 (23.2)
Period of time in	<3 Months	0	0		79 (9.6)	33 (4.0)	335 (40.8)
mental health	3–6 Months	2 (0.2)	80 (9.7)		219 (26.6)	195 (23.8)	127 (15.5)
services at first	7–9 Months	172 (21.0)	196 (23.9)		186 (22.6)	159 (19.4)	75 (9.1)
IAPT session	10–12 Months	55 (6.7)	175 (21.3)		250 (30.4)	138 (16.8)	71 (8.6)
	12+ Months	592 (72.1)	370 (45.1)		88 (10.7)	296 (36.1)	213 (25.9)
Psychotropic medication	Use at baseline	397 (48.4)	412 (50.2)		429 (52.2)	429 (52.3)	464 (56.5)
Referral source	GP referral	617 (75.2)	677 (82.5)		684 (83.2)	798 (97.2)	771 (93.9)
	Self-referral	81 (9.9)	102 (12.4)		84 (10.2)	8 (1.0)	4 (0.5)
	Any specialty	94 (11.4)	27 (3.3)		14 (1.7)	6 (0.7)	17 (2.1)
	Other	29 (3.5)	15 (1.8)		40 (4.9)	9 (1.1)	29 (3.5)
PCT	A	560 (68.2)	176 (21.4)		70 (8.5)	1 (0.1)	0
	B	0	0		0	122 (14.9)	571 (69.5)
	C	0	0		0	191 (23.3)	89 (10.8)
	D	158 (19.2)	374 (45.6)		517 (62.9)	27 (3.3)	0
	E	0	0		0	462 (56.3)	161 (19.6)
	F	57 (6.9)	164 (20)		135 (16.4)	9 (1.1)	0
	G	46 (5.6)	107 (13.0)		100 (12.2)	9 (1.1)	0
PHQ-9 score	No Impairment (0–5)	125 (15.2)	67 (8.2)		55 (6.7)	119 (14.5)	93 (11.3)
	Mild Impairment (5–9)	234 (28.5)	142 (17.3)		142 (17.3)	209 (25.5)	178 (21.7)
	Moderate Impairment (10–14)	242 (29.5)	268 (32.6)		218 (26.5)	210 (26.5)	197 (24.0)
	Moderate-Severe Impairment (15–19)	163 (19.9)	226 (27.5)		223 (27.1)	170 (27.1)	207 (25.2)
	Severe Impairment (20–27)	57 (6.9)	118 (14.4)		184 (22.4)	113 (22.1)	146 (17.8)
GAD-7 score	No Impairment (0–5)	125 (15.2)	39 (4.8)		60 (7.3)	116 (14.1)	76 (9.3)
	Mild Impairment (5–9)	242 (29.5)	198 (24.1)		184 (22.4)	222 (27.0)	198 (24.1)
	Moderate Impairment (10–14)	213 (25.9)	282 (34.3)		270 (32.8)	206 (25.1)	265 (32.3)
	Severe Impairment (15–21)	241 (29.4)	302 (36.8)		308 (37.5)	277 (33.7)	282 (34.3)
WSAS score	No Impairment (0–10)	380 (46.3)	241 (29.4)		213 (25.9)	297 (36.2)	196 (23.9)
	Mild Impairment (11–20)	286 (34.8)	334 (40.7)		313 (38.1)	304 (37.0)	324 (39.5)
	Moderate Impairment (21–30)	131 (16.0)	195 (23.8)		243 (29.6)	172 (21.0)	227 (27.6)
	Severe Impairment (31–40)	24 (2.9)	51 (6.2)		53 (6.4)	48 (5.8)	74 (9.0)

		Mean (SD)					
Phobia Measures	Phobia Q1 Mean (SD)	2.2 (2.1)	2.7 (2.4)		3.2 (2.6)	2.6 (2.4)	3.1 (2.6)
	Phobia Q2 Mean (SD)	1.8 (2.3)	2.2 (2.5)		2.4 (2.6)	2.4 (2.6)	2.7 (2.8)
	Phobia Q3 Mean (SD)	1.9 (2.6)	2.1 (2.6)		2.3 (2.7)	2.0 (2.6)	2.2 (2.7)

*Stratum one*, which included those most likely to receive telephone interventions, had the lowest overall age and the highest percentage of women (71%) of all five strata. It also had the lowest average PHQ-9, GAD-7, and WSAS scores in addition to the lowest average scores for each phobia measure. 68% of individuals were employed (full or part-time) and 9.4% of individuals were receiving benefits. The majority of individuals in the stratum were seen 12 months or longer after the service initiated. Slightly less than half received psychotropic medication at baseline. It also contained the lowest proportion of patients referred by GPs (75%). Providers were not proportionately represented across strata.

Compared with stratum one, *stratum two* comprised a slightly older population with a smaller proportion of women (63%). PHQ-9, GAD-7, WSAS scores, and all phobia scores were markedly higher than stratum one. Percentage of individuals in full or part time employment was similar to stratum one (67%), although a higher proportion of individuals received benefits (16.6%). 45% of individuals were seen more than 12 months after the service was initiated. 83% of individuals were GP referrals.

*Stratum three* had age and gender characteristics similar to stratum two (63.6% women) with significantly higher average PHQ-9 scores but comparable GAD-7, WSAS scores. Average phobia scores were higher than strata one and two. 55.7% of individuals were employed (lowest of all strata), and 22.9% of individuals were receiving benefits at baseline. Only 10% of individuals were seen after the service had been active for longer than 12 months. 83.2% of individuals were referred by their GP.

*Stratum four* had the second-oldest age profile and the second-highest proportion of women (64.4%). PHQ-9, GAD-7, and WSAS scores were slightly lower than strata two and three, similar to stratum one. Average phobia measures were similar to stratum two. 65.8% of individuals were in full or part-time employment at baseline; 14.8% were receiving benefits. 97% of individuals in stratum four were referred by GPs, and only 36.1% of individuals were seen after the service had been active for longer than 12 months.

*Stratum five* was most like stratum three in terms of its age and gender distribution (63.3% women); it had the highest PHQ-9, GAD7, and WSAS scores of all. Phobia scores were high, also similar to stratum three. 65.6% of individuals were in part or full-time employment and 23.2% of individuals were receiving benefits at baseline. 25.9% individuals were seen after services had been active for longer than 12 months.

Significant differences in baseline means for PHQ-9 between treatments were observed only in stratum one (t-statistic: 2.34, p = 0.02). No significant baseline differences were observed in GAD-7 and WSAS across treatment groups in any stratum. All group average scores for GAD-7 and PHQ-9 indicated moderate impairment (10–14) while all group average WSAS scores were between 10 and 20, indicating functional impairment.

**Figure 3:**
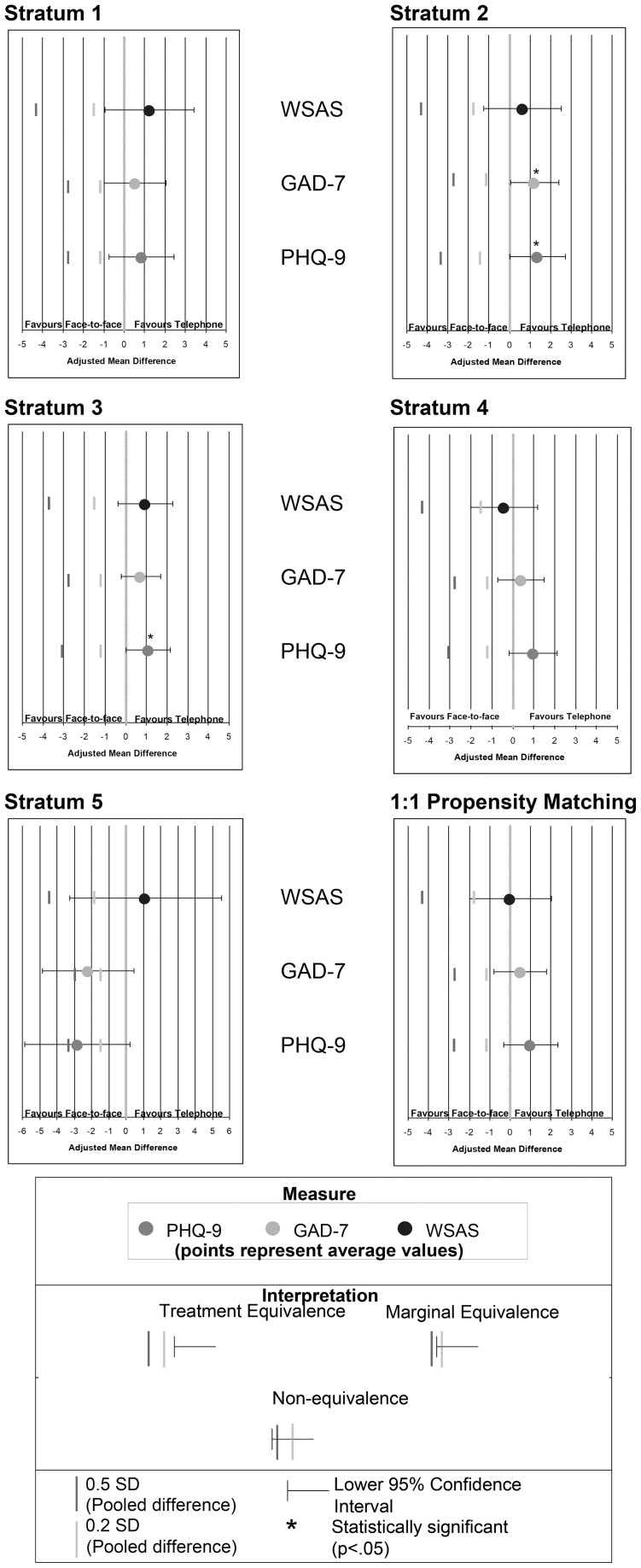
Adjusted mean differences in PHQ-9, GAD-7 & WSAS measures shown as effect sizes. To assess non-inferiority between the treatments we used two-sided significance tests and 95% confidence intervals for score differences between treatment groups. The first pane is a key to the other six that show the results for the five strata (analysis approach 2) and for the 1∶1 matched sample (approach 3). The lower limit of the confidence interval (LCL) represents a boundary of non-inferiority. For all three measures, the LCLs were compared with two estimates of statistical uncertainty: small (0.2x pooled SD; inner vertical line closer to line of equivalence) and medium (0.5x pooled SD; outer line). The next six panes display adjusted mean differences in score reduction between OTT and FTF treatment assessing non-inferiority. In strata one to three, the lower confidence limit (LCL) of the adjusted mean difference fell below 0.2 SD on none of the measures, indicating strong evidence that neither treatment was inferior to the other. In stratum four and in the 1∶1 propensity matching, the WSAS LCL exceeded the 0.2 SD threshold, indicating only marginal support for non-inferiority regarding work and social adjustment improvement. The situation was different in stratum five, the group with most highest symptom scores, where the LCL exceeded 0.2 and 0.5 SD for the PHQ-9 and GAD-7 scores and 0.2 SD for the WSAS. This indicates potentially superior symptom reduction in all domains for individuals receiving FTF CBT. The *a priori* minimally important difference (MID) estimate of 5 points on the PHQ-9 is represented by the extreme limits of the x-axis in Figure 3 (−5 favouring FTF and +5 favouring OTT). No estimates or LCL approached this MID estimate. Furthermore, the effect size is small for all the potential differences, including those reaching statistical significance in strata 2 and 3. See: http://www.psychiatry.cam.ac.uk/wp-content/uploads/2012/08/PONE-D-11-20688-Figures.doc for colour version.

Figure 2 displays the average score reduction within each stratum for all three outcome measures. Figure 3 displays adjusted mean differences in score reduction between OTT and FTF treatment so as to assess non-inferiority. In strata one to three, the lower confidence limit (LCL) of the adjusted mean difference did not fall below 0.2 S.D. on any of the measures; this is strong evidence that neither treatment was inferior to the other. In stratum four (and in the 1∶1 propensity matching, see below) the LCL for adjusted mean difference in WSAS exceeded the 0.2 S.D. threshold, indicating only marginal support for non-inferiority regarding work and social adjustment improvement. The situation was different in stratum five, the group with most severe symptoms. Here, the LCL exceeded 0.2 and 0.5 S.D. for the PHQ-9 and GAD-7 scores and 0.2 S.D. for the WSAS, indicating potentially superior symptom reduction in all domains for individuals receiving FTF interventions.

The minimally important difference (MID) estimate of 5 points on the PHQ-9 [17] is represented by the extreme limits of the x-axis in Figure 3 for this outcome measure (−5 favouring FTF and +5 favouring OTT). No estimates or LCL approached this MID estimate although some statistically differences between OTT and FTF groups were apparent. In strata 2 and 3, reductions in PHQ-9 scores were significantly larger for individuals receiving OTT versus FTF interventions (Stratum two: F: 4.05, p = .045, ES: 0.22, Stratum three: F: 4.09, p = .043, ES: 0.18). The average reduction in GAD-7 scores for those receiving OTT interventions was significantly higher in stratum two (F: 4.20, p = .041, ES: 0.22). Thus, whether they favoured OTT or FTF, any statistically significant effects identified in the comparisons within strata were small to medium (as defined as Cohen's d estimates between 0.10 and 0.25) and, for PHQ-9, below the threshold defined as a minimally important difference.

#### Approach 3: Non-inferiority Comparisons & 1∶1 Matching

The 1∶1 matching procedure produced the following numbers of matched pairs in each PCT: A: 53, B: 21, C: 29, D: 186, E: 77, F: 53, G: 37. The lowest number of matched pairs was 21 (42 individuals) from site B. Thus, 21 matched pairs were repeatedly and randomly selected from the six other PCTs so as to produce, always together with the 21 pairs from site B, 40 samples of 147 pairs (Table 4). All univariate comparisons of matching variables across treatment groups were non-significant (data not presented).

**Table 4:** Treatment characteristics and course in FTF versus OTT groups matched 1∶1 on the basis of propensity scores.

Measure of treatment characteristics & course		Face-to-Face (FTF) n = 147		Over-the-Telephone (OTT) n = 147		Mean *p*-value (95% CI) 40 replications
		N (%)	%	N (%)	%	
Service outcome	Completed treatment	77	52.4	70	47.6	
	Declined treatment	11	7.5	16	10.9	
	Dropped out of treatment	43	29.3	52	35.4	
	Not Suitable for treatment	16	10.9	9	6.1	0.375 (0.296–0.454)
Therapy modality	Computerised CBT	13	8.8	43	29.3	**0.001 (0.001–0.002)**
in patients	Pure Self-Help	94	63.9	87	59.2	0.524 (0.516–0.532)
receiving at least	Guided Self-Help	77	52.4	65	44.2	0.137 (0.080–0.194)
one session	Behavioural Activation	29	19.7	20	13.6	0.387 (0.290–0.484)
	Structured Exercise	27	18.4	11	7.5	0.379 (0.283–0.475)
	Psycho-educational Groups	10	6.8	3	2.0	**0.072 (0.048–0.096)**
Number of	1	61	41.5	77	52.4	
Therapy modalities	2	54	36.7	50	34.0	
received during	3	23	15.6	13	8.8	
treatment course	4+	9	6.1	7	4.8	0.744 (0.690–0.798)
		**Mean**	**SD**	**Mean**	**SD**	
Mean number of treatment sessions		3.88	2.03	3.93	2.11	0.844 (0.549–0.922)
Mean total treatment duration (hours:minutes, SD in mins.)		03:27	159	02:20	84	**<0.001 (<0.001–0.210)**

Note: Values in bold indicate significant associations (p<.01).

Baseline PHQ-9 and GAD-7 mean scores for both OTT and FTF interventions indicated moderate depression and anxiety (PHQ-9: 13.0 and 11.6, GAD-7: 12.0 and 10.9 for OTT and FTF, respectively). WSAS baseline means indicated functional impairment (16.17 and 14.76, respectively). No significant difference in the baseline average values across groups was observed. (PHQ-9 *t*-score: −1.91, *p* = 0.06, GAD-7 *t*-score: −1.625, *p* = 0.11, WSAS *t*-score: 1.29, *p* = 0.20).

Figure 2 displays the average score reduction in the 1∶1 matching sample for all three measures (bottom pair of bars in each panel). The bottom right pane in Figure 3 summarises adjusted differences in treatment outcomes between OTT and FTF treatment administration in the 1∶1 matched sample. WSAS exceeded the 0.2 S.D. LCL, indicating marginal equivalence. PHQ-9 and GAD-7 did not, indicating that neither OTT nor FTF was an inferior treatment to the other. As for the within strata comparisons, the lower limit of the confidence interval for the PHQ-9 did not exceed the five point change estimate that would have indicated the possibility of a minimally important difference between scores.

Results from the random-effects ANCOVA revealed no significant difference in the magnitude of reductions in PHQ-9 (OTT: 5.6 (6.4), FTF: 4.8 (5.3)), GAD-7 (OTT: 5.1 (6.0), FTF: 4.8 (5.3)), and WSAS (OTT: 5.4 (9.1), FTF: 5.7 (8.2)); (PHQ-9 *F*: 2.185, *p* = 0.140, GAD-7 *F*: 0.576, *p* = 0.449, WSAS *F*: 0.004, p = 0.994).

#### Treatment characteristics and course in the FTF and OTT groups matched 1∶1 on the basis of propensity scores

Table 4 provides information on the type, duration, and content of treatments provided in both treatment groups derived from the sensitivity analysis (random draws). For all outcomes, reported proportions were taken from a single, representative random draw while significance levels were calculated using iterative re-sampling of matched pairs.

No significant difference was observed across groups in terms of type of treatment outcomes (average χ^2^: 3.73, average p = 0.375, 95% CI: 0.296–0.454, indicating no difference in the manner in which patients terminated treatment across groups. Those receiving OTT interventions were significantly more likely to receive computerised CBT (average χ^2^: 16.30, average *p*<0.001, 95% CI: 0.001–0.002). Those receiving OTT interventions were less likely to receive psycho-educational group interventions, although the difference was marginal (average χ^2^: 4.565, *p*<0.005, 95% CI: 0.048–0.096). All other treatment type comparisons were non-significant. No significant differences across groups in the number of modalities received during the course of treatment were observed (average χ^2^: 1.27, *p*<0.005, 95% CI: 0.690–0.798).

No significant difference was observed in the number of treatments received (average *t*-statistic: 0.2, *p* = 0.84, 95% CI: 0.55–0.80), although a significant difference in the total duration of treatment was observed (average *t*-statistic: 3.74, *p*<0.001, 95% CI: <0.001–0.210). On average, individuals receiving OTT treatment received 32.6% less contact time than equivalent FTF patients.

### Cost-minimisation Analysis

Under the assumption that OTT costs 32.6% (approx. 1.5 times) less than FTF, the cost per session was estimated for a base-case scenario (Table 5). The mean cost per session for OTT was £79.19 (95% CI 55.0 to 103.3) and FTF was £ 118.76 (95% CI 82.5 to 155.0). Even when the cost ratio was varied from 1.2 times to 2 times, OTT was still cost-effective.

**Table 5:** Cost per session for over-the-telephone (OTT) and face-to-face (FTF) therapies in IAPT during 2009–2010.

PCT	Total IAPT Spend (£)	Total Spend on Low Intensity	Total Costs		Total Sessions		Mean Session Cost (£)	
		Sessions (£)	OTT	FTF	OTT	FTF	OTT	FTF
A	2,029,000	752,835	416,462	336,373	5432	2925	76.67	115.01
B	2,188,000	978,790	53,632	925,158	867	9971	61.86	92.79
C	2,521,000	829,835	124,921	704,914	1160	4365	107.65	161.48
D	4,050,000	1,269,143	447,933	821,210	7293	8914	61.42	92.13
E	2,647,000	984,611	125,695	858,916	1424	6488	88.26	132.39
**Mean Session Cost (95% CI)**							79.19 (55 to 103)	119 (83 to 155)

## Discussion

This comparison of talking therapies delivered OTT or FTF was a naturalistic study in established, low intensity IAPT services across an entire region of England; the sample size is large and uses routine, systematic and prospectively collected patient-reported outcomes. Existing evidence for the effectiveness of talking therapies based on CBT for mild to moderate depression and anxiety is strong and based on gold-standard randomised controlled trials (RCTs); the same is true for therapy delivered over the telephone. However, these trials are predominately in the context of small samples from research-based clinical settings. On the basis of Government mandate, IAPT services have been introduced at great pace throughout England, negating the possibility of extending the evidence from RCTs to the question as to whether CBT or its mode of delivery (e.g. FTF or OTT) are clinically- or cost-effective when scaled-up from the clinical laboratory to the entire population. In these circumstances, where even cluster randomisation is impossible, a range of further methodologies must be deployed in order to assess the comparative effectiveness of health services [22]. The results of observational studies need to be interpreted with great care because of potential bias and confounding. Nevertheless, it is important to exploit the information from routinely collected information in order to inform policy makers, clinicians and patients in the common circumstances that are beyond randomised trials.

In this vein, we have carefully applied a series of analytical approaches to the question of comparative effectiveness in common mental disorders of CBT delivered face-to-face or over-the-telephone in a network of health services that are already in place. We believe that some conclusions can be drawn and recommendations made for further work. The first analytical approach, a naïve comparison between is presented for completeness and in order to show how findings develop and the approach becomes more sophisticated. The following propensity score approaches militate against bias and confounding resulting from selection to treatment effects such as the systematic use of one treatment modality or another according to characteristics of the particular IAPT service or individual participants.

The two propensity score approaches compared the efficacy of the two treatments in individuals who were similar in their baseline treatment profiles and controlled for systematic differences between providers of the IAPT service. We demonstrated that OTT and FTF showed equivalent effectiveness for anxiety symptoms (GAD-7), depression symptoms (PHQ-9), and work and social functioning (WSAS) in all but the most severely affected patients who were identified within stratum five. Initial, unadjusted comparisons suggested OTT was actually more effective than a FTF approach but these results were heavily contaminated by selection to treatment effects and should be discounted in favour of results from the propensity approaches.

### Strengths and Limitations of the study

Major strengths include the regional setting across several services, use of routine PROs and the large number of covariates available for the propensity score. Despite exclusions due to lack of treatment and missing data, the sample is much larger than comparable RCTs that often have sample selection effects, limited generalizability and unsatisfactory comparison groups such as waiting list controls. Subjects were inevitably excluded from the propensity sampling but each individual stratum and the 1∶1 matched sample amount to some of the largest samples ever used to assess the effectiveness of telephone based interventions in routine psychological care. Reliance on comparisons with individuals on a waiting list and seen in routine GP care are a recognised weakness in the current literature assessing telephone based psychological interventions [6]. Our results are widely applicable given that the sample was drawn from a range of different IAPT providers implementing different service structures and treatment models, with individual participants representing a broad cross-section of the population within an English region.

The study has limitations. We cannot be certain whether the benefits seen across both groups were genuine effects of the IAPT treatment or were due to natural resolution of symptoms and regression to the mean. However, the CBT interventions have been shown to have efficacy in well-designed RCTs so it is a reasonable assumption that there were real treatment effects to be compared. There may be a degree of hidden bias and residual confounding due to un-assessed covariates (including unimagined factors) that would have been equalised in a randomised design; patient safety, mobility and previous illness history represent possible factors here. We see the rating of the outcomes by patients, themselves, as a positive aspect of the study. The measures were used in the same way in the two treatment arms and neither patients nor clinicians were aware that the comparison of FTF and OTT would subsequently be made. Independent, blind assessments by a third party would have been a useful corroboration but were impractical in an in-service evaluation such as this. The representativeness of the data remains a concern; those individuals who fail to report all of the items on the IAPT minimum dataset may be more (or less) likely to drop-out of treatment or to experience less symptom reduction. This can be better assessed as further data accrue in a wider range of IAPT service system providers.

Another limitation is that the effectiveness of OTT and FTF interventions cannot be assessed according to an exact clinical diagnosis given likely differences in diagnostic accuracy and conventions across the numerous health-care professionals within the sample of providers. All that can be said is that telephone-based interventions are effective in the treatment of many individuals presenting to the lower intensity tier of IAPT services in the East of England. The vast majority of these people will have mixed anxiety and depression, sometimes referred to as common mental disorder. Nevertheless, the accuracy and standardisation of clinician diagnosis and possible differential effects of treatment according to more detailed diagnostic categories remain important areas of future research, albeit that these are unlikely ever to be used consistently on a large scale.

A fifth of potentially eligible subjects were excluded because they received a mixture of FTF and OTT treatments following their baseline assessment. It would have been useful to have included these subjects on the basis of an intention-to-treat analysis. This was not possible because we had no indication of what the intention was; we could have allocated subjects to either OTT or FTF on the basis of the nature of their contact in the session following baseline, but this would have been an assumption of intention that may not have been warranted. It is likely that mixtures of FTF and OTT arise because of the uncertainty as to which may be better, something our study was intended to resolve, or because the true intention was a blend of treatment, a third treatment arm.

The total sample size was large but became restricted as the analysis moved into the propensity strata and the 1∶1 matched sample. Thus, the statistical power needs consideration. We were careful to ground the analyses in the context of conventionally defined effect sizes and, for the measure of depression, the PHQ-9, in an accepted definition of a minimally important difference [17] that equates to a large effect size. Thus, we can be clear from our results that we have not rejected such a treatment difference or greater on the basis of a Type II statistical error; none of the confidence limits in the 1∶1 analysis approached such a difference and a *post-hoc* power analysis indicated that this analysis was sufficiently powered, with conventional parameters, to exclude, had they existed, a large effect for PHQ-9 (ES 0.27) and moderate effects for GAD-7 and WSAS (ES 0.16 and 0.24, respectively).

A limitation of the costing approach adopted is that it was primarily based on assumptions concerning estimates of the relative costs of OTT and FTF treatments. Given that the costs of IAPT are largely the costs of staff and clinical facilities, with education costs being similar in both groups, it is likely that our results based on staff time will be valid, with differences remaining similar even if a more detailed micro-costing approach was to be undertaken. Estimates of cost savings are also incomplete; additional savings in the form of reduced travel and reduced need for clinical accommodation remain important considerations to be incorporated into future calculations. Our view is that these would magnify the differences that we have observed.

### The findings

Our results indicate that symptoms decrease and social function increases under both treatment conditions, and that OTT is a convenient and effective CBT modality for the majority of patients treated within the lower intensity tier of IAPT services. There was an important indication of heterogeneity of effect: the propensity strata showed that those who were older and had higher symptom scores (stratum 5) may do better with FTF. This conclusion has face validity given that these people would be closest to the threshold for the more intensive IAPT therapies given face-to-face that are not the subject of this study that concentrated on lower intensity treatments.

Thus, for the majority, the convenience of OTT to patients and services embeds a likely economic advantage to OTT for most people. The cost-minimisation analysis focused on service costs, alone. It indicated that the cost per OTT session was approximately one third lower than that of a FTF session, a result that was robust when the model assumptions were changed. The delivery of OTT or FTF therapy appeared to depend not on patient characteristics but mainly on where and when the treatment took place. This suggests that it is within the remit of commissioners and services to design IAPT services, accordingly, though further work is needed to define the characteristics of those people with severe disorder, such as those in stratum five, who will, in fact, do better with a FTF model. Overall, the results indicate that increasing the proportion of low intensity talking therapy delivered OTT in some areas may reduce the cost of the IAPT programme, increase its productivity and maintain the quality of the service. Further research is needed to better identify the group of people with more severe illness in stratum 5, initially assessed clinically as appropriate for the lower level therapies; they may have better outcomes with FTF therapy.

#### Comparison with other studies

A recent meta-analysis [20] demonstrated benefits for the use of technology-mediated interventions in the treatment of depression and anxiety, but only two studies compared telephone with face-to-face delivery. One RCT of 72 patients showed that CBT delivered by telephone was equivalent to treatment delivered face-to-face with similar levels of satisfaction.[7]. This study involved OCD sufferers, a specific anxiety disorder, and highly trained therapists akin to those in the higher intensity IAPT tier. A meta-analysis comparing the effect of self-guided interventions with brief therapeutic input, common in the IAPT low intensity setting, found that brief contact did boost effectiveness [10] but FTF or OTT contacts were not compared. Nevertheless, our large-scale, pragmatic evaluation of IAPT in a real-world setting is consistent with the findings of the prior work, strengthening the case for action.

The results from the 1∶1 stratification sample indicated that OTT interventions can provide significant reductions in the total amount of time each patient is seen. Our estimates were somewhat lower than the 40% reported in other published accounts but further efficiencies may be found as services mature (our IAPT services had been established for only 1–2 years) and if more attention is paid to the design of the OTT approach [7]. Layard and colleagues, seminal contributors to the original IAPT model, estimated that the cost of providing a standard course of roughly ten sessions of CBT is £750 or £75 per session [21], an assumed rather than an observed estimate. Our OTT session costs estimates exceed this but still represent a significant route for cost savings and effective, flexible, and readily available treatment.

### Conclusions and Policy Implications

The clinical effectiveness of low intensity CBT-based interventions delivered over-the-telephone was not inferior to those delivered face-to-face in the majority of patients with the common mental disorders of depression and anxiety. A minority of people with more severe illness and who tended to be older appeared to gain more benefit from face-to-face therapy; research is required in order for services to identify them efficiently. For most, CBT delivered over-the-telephone is a cost-effective and probably convenient option, providing the potential for significant financial savings to the IAPT programme in the lower intensity context. Increasing the proportion of low intensity talking therapy delivered over-the-telephone may reduce the cost of the IAPT programme, increase its productivity and maintain the quality of the service.

In a global context, the potential is enormous for spreading access to effective psychological therapies to the millions of people affected by depression and anxiety. As the availability of mobile phone technology in low and middle income countries grows, people now have the potential of having a therapist in their pocket, transcending traditional barriers to the receipt of effective treatments. Should this opportunity be taken we recommend that randomised evaluations are used to evaluate what could potentially amount to another major step forward in care for common mental disorders.
